# Sports Nutrition

**DOI:** 10.1016/j.cdnut.2025.107613

**Published:** 2025-12-09

**Authors:** Jonathan D Buckley, Jess Gwin, Stuart Phillips

**Affiliations:** 1 Alliance for Research in Exercise, Nutrition and Activity (ARENA), School of Allied Health and Human Performance, Adelaide University, Adelaide, Australia; 2 United States Army Research Institute of Environmental Medicine, Natick, MA, United States; 3 Department of Kinesiology, McMaster University, Hamilton, ON, Canada

## Introduction

Nutrition can play a crucial role in sporting performance. However, what constitutes “proper” nutrition is unclear and can vary between events that have differing demands. Macronutrients—carbohydrates and fats—serve as fuels for muscle contraction, whereas protein is important for building and maintaining skeletal muscle and other tissue mass. Micronutrients also play important roles, as does fluid consumption for hydration. There may also be certain supplements that give “an edge” to athletes. As a result, professional and recreational athletes need to carefully consider their dietary choices to optimize performance.

For this Special Issue of *Current Developments in Nutrition* on Sports Nutrition, authors were encouraged to contribute articles to the field. The manuscripts that were submitted represented 3 major topics: *1*) nutrition to improve athletic performance, *2*) nutrition to improve clinical outcomes, and *3*) nutritional knowledge and diet quality among athletes.

## Nutrition and Athletic Performance

Seven articles [[1], [2], [3], [4], [5], [6], [7]] were published on the effects of various nutrients on athletic performance. Four of these articles evaluated the effects of coffee or caffeine on exercise performance [2,[4], [5], [6]], whereas 2 evaluated the effects of polyphenols or anthocyanins [1,7], and the final one evaluated the effects of creatine monohydrate [3].

In general, the 4 studies evaluating the effects of caffeine or coffee on athletic performance reported improvements in aerobic and anaerobic performance. Soegaard et al. [6] showed that consuming 300 mg caffeine improved mean power output during a 20-min cycle time-trial by ∼5% compared with fasting or carbohydrate consumption. Similarly, in male kick-boxing athletes, Saremi et al. [5] showed that doses of 3 mg/kg and 6 mg/kg consumed 60 min before testing improved treadmill run time to exhaustion during the Bruce Protocol by ∼6% compared with placebo and improved vertical jump performance by ∼5%, but performance improvements did not differ between the 3 mg/kg and 6 mg/kg doses. This finding suggests that a dose of 3 mg/kg may be optimal for improving endurance and power performance, but the 6 mg/kg dose resulted in less muscle soreness than the placebo or the 3 mg/kg dose 2 h postexercise. Heydari et al. [2] evaluated the effects of postactivation performance enhancement (PAPE) and caffeine supplementation (6 mg/kg) on explosive and anaerobic power in recreationally active males. They assessed effects on vertical jump height, standing long jump, sprint performance (40-yard dash) and the running-based anaerobic sprint test (RAST). They found that, apart from for the standing long jump, caffeine and PAPE both improved performance, with effects of caffeine and PAPE being similar, but for vertical jump height, there was a synergistic effect such that performing PAPE with caffeine supplementation resulted in a greater improvement in performance than PAPE or caffeine alone. In male basketball players, Niknam et al. [4] evaluated the effects of coffee consumption (low-dose espresso coffee [80 mg caffeine], high-dose espresso coffee [160 mg caffeine], decaffeinated espresso coffee placebo [10 mg caffeine], or no-coffee control) on repeat sprint performance, perceived fatigue, and eye-hand coordination (soda pop test). They found that coffee dose-dependently improved repeat sprint performance compared with placebo and control and reduced perceived fatigue after the repeat sprint exercise. They also found that, prior to and immediately after the repeat sprint exercise, performance on the soda pop test improved with both coffee and placebo, indicating improved eye-hand coordination, but only due to a placebo effect. However, immediately after and 5 min after the repeat sprint exercise, performance on the soda pop test improved in a dose-dependent manner compared with placebo, indicating that, although there was a placebo effect, the larger effect was due to the dose of coffee consumed. Taken together, these studies suggest that caffeine supplementation and/or coffee consumption can improve various aspects of aerobic and anaerobic exercise performance, mitigate perceptions of fatigue, and improve eye-hand coordination. These benefits would provide an advantage in a range of individual and team sports.

Two articles evaluated the effects of polyphenols [1] and anthocyanins [7], which are a subclass of polyphenols, on exercise performance and/or recovery. The study that evaluated the effects of polyphenols on exercise performance [1] had recreationally trained cyclists consume a mix of polyphenol-rich almonds, dried grapes, and cranberries or an isocaloric oat bar control daily for 5 wk during a run-in period of light training followed by 2 wk of heavy training and then a 2-wk taper period. A 5-min cycling time-trial was performed at the end of each training period. Compared with the end of the run-in period, cycle time-trial performance was reduced (due to fatigue) at the end of the heavy training period and improved at the end of the taper period, but with no difference between participants who consumed the polyphenols and those who consumed the oat bar control. However, participants who consumed polyphenols demonstrated increased nitric oxide bioavailability and improved subjective energy measures. The article that evaluated the effects of anthocyanins on exercise performance and recovery was a narrative review that focused primarily on the effects of intake of anthocyanin-rich black currant supplements [7]. The review included 17 studies and found that 60% of studies evaluating effects on exercise performance reported positive effects, whereas 78% reported benefits for recovery, but noted that the mechanistic understanding of the beneficial effects remains limited.

Meixner et al. [3] performed a placebo-controlled crossover study in 20 male and 5 female cyclists to evaluate the effects of creatine monohydrate (4 × 5 g/d) on sprint performance and found improvements in 15-s maximal work output that appeared to be mediated by an increase in fat-free mass.

Thus, taken together, these papers suggest that caffeine and creatine monohydrate can be effective for improving exercise performance, whereas results for polyphenols/anthocyanins are mixed.

## Nutrition and clinical outcomes

Five articles were published that outlined the effects of nutrition on various sport-related clinical outcomes [[8], [9], [10], [11], [12]]. Of these, 2 studies were focused on the potential role of n–3 polyunsaturated fatty acids in ameliorating effects of head impacts/brain injury/concussion [11,12], 1 study evaluated associations between dietary protein intake and markers of bone health in endurance athletes [10], and a fourth study evaluated the use of the resting metabolic rate ratio as an indicator of risk of Relative Energy Deficiency in Sports (RED-S), which brings with it a risk of poor bone health. The last of the 5 studies evaluated nutritional strategies employed by dietitians and nutritionists in Ireland to promote recovery from injury and return to play [8].

Heileson et al. [11] performed a meta-analysis of data from 3 studies in American football players that evaluated effects of long-chain n–3 (LCn-3) supplementation on neurofilament-light (Nf-L), which is a brain-derived biomarker of neuroaxonal injury that has been shown to be elevated in response to repetitive subconcussive head impacts in contact sports. The authors found that compared with placebo, long-term supplementation with LCn-3 was associated with a smaller increase in Nf-L by the end of the competitive season, providing preliminary support for the prophylactic administration of LCn-3 in contact sport athletes who are exposed to repetitive subconcussive head impacts. Similarly, Lust et al. [12] performed a dose-response study of LCn-3 in male mice who had a mild traumatic brain injury induced after 5 wk of consuming a high LCn-3, moderate LCn-3, or a control diet. They found that mice that consumed the diet high in LCn-3 had significantly improved functional performance (righting reflex and time to seek) at 4 h post brain injury compared with those who consumed the control diet. Taken together, the findings of the studies by Heileson et al. [11] and Lust et al. [12] suggest that consuming LCn-3 may confer neuroprotective benefits against brain injury for athletes competing in sports that might expose them to potential brain injuries.

Endurance athletes are at increased risk of compromised bone health due to high-volume training, which increases nutritional demands. In female athletes, if energy intake is insufficient relative to demand, RED-S can develop, which, among other consequences, results in compromised bone health. Garay et al. [9] performed a cross-sectional analysis of female recreational and collegiate athletes and found that 63% of participants exhibited low energy availability, which predisposes to the risk of RED-S. However, although energy availability is typically used to identify risk of RED-S, they found that the resting metabolic rate ratio, calculated as the measured resting metabolic rate (by indirect calorimetry) divided by the resting metabolic rate calculated using prediction equations, was a more sensitive indicator of RED-S risk than energy availability. In another cross-sectional study in both male and female athletes, Gardy et al. [10] found that while total dietary protein intake was weakly associated with higher lumbar spine areal bone mineral density, protein intake from animal products was more strongly and positively related to estimated bone strength of the tibia and tibial cortical volumetric bone mineral density. Thus, identifying female athletes with RED-S may be evaluated using the resting metabolic rate ratio, and once identified, dietary interventions to increase animal protein intake may be beneficial.

In the final article in the Special Issue that evaluated dietary influences on clinical outcomes, Finnegan et al. [8] evaluated nutritional strategies employed by dietitians and nutritionists in Ireland to promote recovery from injury and return to play. They found that there was considerable diversity in the nutritional strategies employed across sports, organizations and genders, which influenced athletes’ recovery outcomes. Some of the diversity in strategies employed was a result of contextual factors such as the quality of communication and interdisciplinary collaboration within each particular sporting environment, and the authors proposed implementing evidence-based guidelines, using consistent assessment tools, and enhancing multidisciplinary collaboration to reduce inconsistencies and improve outcomes for athletes.

## Nutritional Knowledge and Diet Quality

Six articles were published on various aspects of nutritional knowledge and diet quality in athletes [[13], [14], [15], [16], [17], [18]]. Two studies evaluated methods used to convey information related to sports nutrition [16,17]. Deslippe et al. [16] used semistructured interviews and thematic analysis to evaluate where adolescent athletes and nonathletes obtained information regarding nutrition. They found that most information came from social media, but that athletes valued more credible sources of information than did nonathletes and that athletes viewed food more as a source of fuel whereas nonathletes viewed food more as an opportunity to form social bonds through eating together. In a related study, Kiss et al. [17] evaluated the nutritional information and communication methods used by nutrition experts and nonexperts in sports nutrition videos on the social media platform, YouTube, which they reported has become a key source of nutritional information for athletes. They identified 4 themes in the nutrition information provided, including the function of nutrition in sports, know-how, dietary strategies, and developing a dietary framework. However, they also identified differences in communication methods between nutrition experts and nonexperts, with expert videos often lacking the communication techniques that nonexperts used to build trust and connect with viewers. They proposed that there was a need for experts to use more effective strategies to engage viewers and build trust and that this might be achieved through collaboration between experts and nonexperts.

Three articles focused on food choices and diet quality in athletes [14,15] and military women [18]. Carey et al. [15] evaluated factors influencing food choice among athletes and active populations in Ireland and found that sensory appeal was the predominant influence, with awareness of a food’s effect on health and performance also having a considerable impact, whereas food values and beliefs had the least impact. Barney et al. [14] evaluated dietary intake and diet quality during a competitive season among female and male National Collegiate Athletic Association Division 1 cross-country running student-athletes at Florida State University (United States). They found that the athletes consumed inadequate carbohydrates, calcium, and vitamin D but met or exceeded intake guidelines for protein, fat, and iron, and their overall diet quality was poor. In contrast, Oliver et al. [18] reported that elite female warfighters and female graduates of combat training from Fort Sill in Oklahoma (United States) exhibited good diet quality scores compared with civilian women and had reduced biomarkers of cardiometabolic disease risk (lipid profile, glucose, and insulin). Based on these findings, the authors suggested that the military nutrition environment promoted female warfighter health.

Bajool et al. [13] investigated the nutritional knowledge and prevalence of dietary supplement use among Iranian bodybuilders. The authors reported that dietary supplement use was greater among male bodybuilders than among female bodybuilders and was greater among athletes with a longer history of bodybuilding and among those who performed more exercise sessions per week. The most common dietary supplements used were vitamin C (used by 56% of participants), vitamin D (50%), whey protein (46%), branched-chain amino acids, and caffeine (both 34%). Bodybuilders sourced most nutrition information from fitness coaches and registered dietitians/nutritionists, and they consumed supplements to improve performance, meet the increased nutritional demands of exercise, and prevent injury and improve recovery. However, concerningly, the bodybuilders reported commonly experiencing adverse side effects of consuming dietary supplements, including skin rashes, elevated liver enzymes, and hair loss.

Thus, it seems that much information regarding sports nutrition is gleaned from social media by athletes, but although some athletes (e.g. female warfighters) have good dietary habits and healthy diets, others have relatively poor diet quality, with dietary choices being influenced to some extent by possible effects on athletic performance but to a greater extent by sensory appeal. Bodybuilders in particular consume dietary supplements at relatively high rates in an effort to improve performance, but some of the supplements consumed can have adverse effects on their health.

In summary, this Special Issue of *Current Developments in Nutrition*, which focused on Sports Nutrition, has identified nutritional strategies that can be used to improve athletic performance and mitigate adverse clinical outcomes associated with injury and has identified key sources where athletes obtain nutrition information and strategies that might be useful to improve the quality of delivery of that information. We therefore believe that this Special Issue will make an important contribution to advancing our understanding of a range of factors related to improving Sports Nutrition outcomes.

## Funding

No funding was provided to support the preparation of this Editorial.

## Conflict of interest

JDB is an Editorial Board Member for *Current Developments in Nutrition* and played no role in the Journal’s evaluation of the manuscript. JG and SP were Guest Editors for *Current Developments in Nutrition* for the Special Issue on Sports Nutrition. The opinions or assertions contained herein are the private views of the authors and are not to be construed as official or as reflecting the views of the United States Army or United States Department of Defense. Any citations of commercial organizations and trade names in this report do not constitute an official Department of the Army endorsement of approval of the products or services of these organizations.
